# Comparison of the Localizer (RFID) and Wire Localization (WL) Techniques in the Localization of Non-palpable Breast Tumors: A Retrospective Cohort Study

**DOI:** 10.7759/cureus.91052

**Published:** 2025-08-26

**Authors:** Eva Koulaymi, Charbel El Rahi, Berengere Tate, Louise Bro De Comeres, Vincent Villefranque, Elie Mikhael

**Affiliations:** 1 Obstetrics and Gynecology, Hôpital Simone Veil, Eaubonne, FRA

**Keywords:** breast cancers, breast conserving, breast oncology, general surgery and breast cancer, malignant breast tumors

## Abstract

Introduction

Since the introduction of breast cancer screening programs through mammography, the number of breast tumors requiring surgery has increased, particularly non-palpable tumors. Surgery on non-palpable breast tumors necessitates the use of image-guided preoperative localization methods. These are divided into wire methods, such as Wire Localization (WL), and wireless methods such as the Localizer (radiofrequency identification; RFID). Each has its own advantages and disadvantages. The objective of this study was to compare margin status, re-excision rates, operative time, and device migration between Localizer (RFID) and WL in the surgical management of non-palpable breast tumors.

Material and methods

A cross-sectional retrospective cohort study was conducted on patients who underwent partial mastectomy for non-palpable breast tumors between January 1, 2020, and December 31, 2023, at Simone Veil Hospital, Eaubonne, France. Patients were divided into two groups according to the tumor localization technique used. Univariate analyses were performed to assess the relationships between the two groups regarding patient characteristics, surgical parameters, and surgical outcomes.

Results

Out of 129 patients, 66/129 (51.1%) underwent tumor localization with the Localizer, while 63/129 (48.8%) underwent tumor localization with WL. The rate of positive margins (RFID: 12/66 (18.2%) versus WL: 18/63 (28.6%), p = 0.23), re-excision for positive margins (RFID: 9/66 (13.6%) versus WL: 15/63 (23.8%), p = 0.21), and operative complications (RFID: 14/66 (21.2%) versus WL: 9/63 (14.3%), p = 0.43) were similar between the two groups. The period between device insertion and surgery was 9 (± 15.8) days for the Localizer and 1 (± 0.5) days for WL (p < 0.01). The operative time was 81.1 (± 35.9) minutes when WL was used, compared to 67.9 (± 27.8) minutes for the Localizer (p = 0.02). The migration rate of the device was comparable between the two methods (RFID: 4/66 (6.1%) versus WL: 3/63 (4.8%), p = 1). Age (RFID: 59.9 (± 14.1) versus WL: 62.9 (± 14.3), p = 0.024) and device migration (OR = 1.7, p = 0.04) were associated with margin status.

Conclusion

In this retrospective cohort, no statistically significant differences were observed between the Localizer and WL in terms of surgical margins or complications. The Localizer, however, provided logistical advantages, including earlier placement and shorter operative time. Importantly, older age and device migration were significantly associated with positive margins, offering clinically relevant insight for surgical planning. Larger prospective or randomized trials are required to confirm whether meaningful differences exist between these localization methods, after which patient-centered outcomes, such as comfort and acceptability, should be further explored.

## Introduction

Breast cancer is the most common type of cancer among women, affecting approximately one in eight women over the course of their lifetime. This corresponds to about 60,000 new cases diagnosed annually in France, or nearly 1,200 new cases per day. Breast cancer remains the leading cause of death among women aged 35 to 55, with the mortality rate projected to reach 162,400 deaths in 2023 in France [1-3].

Since the advent of screening mammography, an increasing number of breast tumors requiring surgery are non-palpable. Surgical excision of non-palpable breast tumors requires the use of an image-guided preoperative localization method [4]. There are several techniques for localizing non-palpable breast tumors. The two most commonly used methods are Wire Localization (WL) and wireless methods utilizing an RFID chip, known as the Localizer [1,4].

Traditional WL methods present disadvantages for both patients and healthcare logistics. With advancements in technology, wireless localization methods appear to offer significant advantages over WL methods, and this warrants further investigation.

Locating non-palpable breast tumors has long been a challenge for surgeons and radiologists, particularly due to issues such as clip migration or stereotactic displacement of the hook-wire. An ideal solution would be a tool that can be easily located without the need for stereotactic intervention. It is suggested that the Localizer may be more effective and accurate than WL in identifying and excising non-palpable breast tumors.

The primary objective of this study is to compare the Localizer (RFID) and WL in the surgical management of non-palpable breast tumors, focusing on margin status, re-excision rates, operative time, and device migration. In addition, the study evaluates potential advantages in workflow efficiency and surgical logistics.

## Materials and methods

We conducted a retrospective cohort study of patients who underwent partial mastectomy for non-palpable breast tumors at Simone Veil Hospital, Eaubonne, France, between January 1, 2020, and December 31, 2023. Eligible patients were identified by cross-checking surgical coding lists with pathology records to ensure comprehensive inclusion. Inclusion criteria were: age ≥ 18 years, a diagnosis of non-palpable breast tumor, and localization performed with either the Localizer (RFID) or WL. Patients were excluded if they presented with palpable tumors, had a prior history of surgery or radiotherapy to the affected breast, or had contraindications to either localization method. Data were collected retrospectively from electronic medical records, including demographic information, tumor characteristics, surgical variables, and postoperative histological outcomes. Cases with incomplete data for primary outcomes were excluded.

A total of 129 patients were divided into two groups based on the preoperative localization method: Localizer RFID tag or WL. WL was performed under ultrasound or stereotactic guidance in the Radiology Department. Using an aseptic technique, a hooked wire was inserted percutaneously into the breast so that the tip lay within or immediately adjacent to the lesion. Positioning was confirmed mammographically. The external portion of the wire was secured to the skin surface with a sterile dressing to minimize displacement. Surgery was performed the same day or within 24 hours of wire insertion.

The Localizer, a wireless technology, uses a 9 mm tag implanted in the breast, transmitting a radio signal that can be detected by a handheld probe with a range of 4 cm within tissue [5]. Localization was performed using the aseptic technique under ultrasound or stereotactic guidance, with mammographic confirmation, at the Radiology Department of the hospital, under an experienced radiologist.

The primary outcome was to compare the rate of positive margins and re-excisions between the two methods. Quantitative data were summarized using mean, standard deviation, and interquartile range, while categorical data were presented as percentages.

Given the limited number of events (positive margins, device migration), multivariate modeling was deemed at high risk of overfitting. Therefore, we restricted our analysis to univariate associations.

Univariate analyses were conducted using the chi-squared test, Student's t-test, Welch’s t-test, and Fisher’s exact test as appropriate, with significance set at p < 0.05. Statistical analyses were performed using R software, version 4.1.1 (R Core Team (2023). R: A Language and Environment for Statistical Computing. R Foundation for Statistical Computing, Vienna, Austria).

## Results

A total of 129 patients were included in the study, with a mean age of 61.4 (±14.2) years. Concerning the tumors, 48/129 (37%) were invasive, and the most common breast density was Type B (BI‑RADS (Breast Imaging-Reporting and Data System) classification: scattered fibroglandular densities), present in 48/129 (37.2%) of patients. Detailed patient characteristics are shown in Table 1.

**Table 1:** Characteristics of the population according to the method of tumor localization RFID: Radiofrequency Identification, WL: Wire Localization, BMI: Body Mass Index, df: Degrees of Freedom

	Localizer (RFID)	Wire (WL)	P-Value	Test Used	Effect Size (Cramér's V)
Number	66	63			
Age (years)					
Mean (SD)	59.9 (14.1)	62.9 (14.3)	0.236	Student’s t-test	-
BMI (kg/m2)					-
Mean (SD)	27.2 (5.62)	26.7 (5.97)	0.658	Student’s t-test	-
Missing	15 (22.7%)	7 (11.1%)			-
Breast density					
A	4 (6.1%)	3 (4.8%)	1	Fisher Exact Test	-
B	16 (24.2%)	32 (50.8%)	0.003	Chi-square (df=1)	0.259
C	8 (12.1%)	20 (31.7%)	0.013	Chi-square (df=1)	0.219
D	3 (4.5%)	5 (7.9%)	0.486	Fisher's exact test	-
Missing	35 (53.0%)	3 (4.8%)	4.2×10-^10^	Fisher's exact test	-
Histological type					
Invasive alone	25 (37.9%)	23 (36.5%)	1	Chi-square (df=1)	0.0
In situ alone or associated with invasive	41 (62.1%)	40 (63.5%)	1	Chi-square (df=1)	0.0
Size of the lesion (mm)					
Mean (SD)	16.4 (11.1)	14.7 (12.2)	0.403	Student’s t-test	-
Time between insertion and surgery (days)					
Mean (SD)	9.06 (15.8)	1.05 (0.551)	<0.001	Welch’s t-test	-

Of the 129 patients, 66 (51.1%) underwent localization using the Localizer (RFID), while 63 (48.8%) underwent WL. The main characteristics of the population by tumor localization method are shown in Table 2. No statistically significant differences were observed between the groups in terms of age (RFID: 59.9 ± 14.1 years vs. WL: 62.9 ± 14.3 years, p = 0.23), BMI (RFID: 27.2 ± 5.62 kg/m² vs. WL: 26.7 ± 5.97 kg/m², p = 0.65), breast density (predominantly Type B: RFID: 24.2% vs. WL: 50.8%, p = 0.55), histological type (invasive: RFID: 37.9% vs. WL: 36.5%, p = 1), or lesion size (RFID: 16.4 ± 11.1 mm vs. WL: 14.7 ± 12.2 mm, p = 0.40). However, there was a significant difference in the period between insertion and surgery (RFID: 9 ± 15.8 days vs. WL: 1 ± 0.5 days, p < 0.01).

**Table 2:** Surgery characteristics and outcomes according to the method of localization RFID: Radiofrequency Identification, WL: Wire Localization, df: Degrees of Freedom

	Localizer (RFID)	Harpon (WL)	P-Value	Test Used	Effect Size (Cramér's V)
Number	66	63	-	-	-
Operative time (minutes)					
Mean (SD)	67.9 (27.8)	81.1 (35.9)	0.025	Student’s t-test	-
Missing	1 (1.5%)	5 (7.9%)	-	-	-
Migration of the device	-	-	1	Fisher Exact Test	-
none	62 (93.9%)	60 (95.2%)	-	-	-
Yes	4 (6.1%)	3 (4.8%)	-	-	-
Margin status	-	-	0.235	Chi-square (df=1)	0.10
Negative	54 (81.8%)	45 (71.4%)	-	-	-
Positive	12 (18.2%)	18 (28.6%)	-	-	-
Re-excision for positive margin	-	-	0.21	Chi-square (df=1)	0.11
None	57 (86.4%)	48 (76.2%)	-	-	-
Yes	9 (13.6%)	15 (23.8%)	-	-	-
Postoperative complications	-	-	0.43	Chi-square (df=1)	0.07
None	52 (78.8%)	54 (85.7%)	-	-	-
Yes	14 (21.2%)	9 (14.3%)	-	-	-

Table 2 presents the surgical characteristics and outcomes based on the localization method. A significant difference was observed in operating time (p = 0.025), with a longer duration when WL was used (RFID: 67.9 ± 27.8 min vs. WL: 81.1 ± 35.9 min, p = 0.02). However, no significant differences were found between the groups regarding device migration (RFID: 6.1% vs. WL: 4.8%, p = 1), negative margins (RFID: 81.8% vs. WL: 71.4%, p = 0.23), re-excisions for positive margins (RFID: 13.6% vs. WL: 23.8%, p = 0.21), or surgical complications (RFID: 21.2% vs. WL: 14.3%, p = 0.43).

Age and device migration were significantly associated with margin status, with older patients being more likely to have positive margins (negative margin: 59.9 ± 14.4 years vs. positive margin: 66.3 ± 12.8 years, p = 0.024; OR = 1.7, p = 0.03). BMI (negative margin: 26.9 ± 5.98 kg/m² vs. positive margin: 27.0 ± 5.26 kg/m², p = 0.89), breast density (p = 0.87), duration between device insertion and surgery (p = 0.93), histological type (p = 0.47), lesion size (p = 0.74), and operative time (p = 0.38) were not significantly associated with positive margins.

## Discussion

In the treatment of breast cancer, studies have demonstrated that partial mastectomy combined with adjuvant radiotherapy is as effective as mastectomy, which is why most women opt for conservative surgery with adjuvant treatment. Approximately one-third of breast cancers are non-palpable at the time of diagnosis, making the localization of these tumors crucial. While WL has been historically successful, it has limitations, including wire displacement, patient discomfort, vasovagal episodes, and the need for same-day or day-before localization.

Our study assessed the performance of two localization technologies--wire-based and wireless--in the context of non-palpable breast tumors operated on by three surgeons over four years. We found no statistically significant differences between the two methods in terms of positive margins, re-excision rates, device migration, or surgical complications, with both groups being comparable in terms of age, BMI, breast density, tumor size, and histological type. Importantly, our study was not designed as an equivalence or non-inferiority trial, and the absence of statistically significant differences should not be interpreted as proof that the methods are equivalent. The possibility of type II error due to limited sample size must be considered.

The only notable difference was in operative time, with procedures using WL taking longer. The simplicity and precision of the Localizer, particularly its ability to indicate the distance between the RFID tag and the probe, likely contributed to this result. Previous studies, such as those by DiNome ML et al. and Dauphine et al., have reported improved localization and fewer positive margins with the Localizer [4,5]. Heindl et al. also reported a similar re-excision rate of 13% for the Localizer [1]. The ability of the Localizer to display the real-time distance between the tag and probe allows for more precise margin adjustments during surgery, as confirmed by Cullinane CM et al., Malter W et al., and Parisi S et al. [6-8].

A Cochrane review by Chan BK et al. in 2015 found no clear differences between various radio-guided methods for non-palpable breast lesion localization in terms of clip success, negative margins, or re-excision rates [9]. However, the review emphasized that available studies were small and heterogeneous, limiting the strength of its conclusions and underscoring the need for larger, better-designed trials. Our findings are consistent with this uncertainty, as both techniques in our cohort appeared comparable, but definitive conclusions regarding superiority cannot be drawn. Similarly, Wang GL et al.'s evaluation of 3,879 patients revealed no significant difference between the Localizer and WL in terms of surgical margins and re-excisions [10].

McGugin et al. also found no difference in operative time or re-excisions between the two methods, though they did note some variability based on lesion type [11]. The operative time for ductal carcinoma in situ was longer with the Localizer, possibly due to the learning curve, but this may decrease with experience.

Our study also highlighted a key advantage of the Localizer: the decoupling of localization and surgery, as the Localizer can be inserted well in advance, allowing proper fixation through reactive fibrosis. This flexibility, already approved by the FDA for long-term use [12], improves scheduling, patient care, operating room efficiency, and comfort compared to WL, which is typically inserted the day before surgery.

Despite our inability to demonstrate associations between BMI, breast density, time between device insertion and surgery, histological type, lesion size, or operative time with positive margins, we did find a significant relationship between age, device migration, and margin status. While age is sometimes noted as a factor in margin outcomes, this relationship is inconsistent across studies. Gooch JC et al. found a significant correlation between older age and positive margins [13], but Voguet et al. suggested younger patients (<45 years) may have higher rates of positive margins due to more aggressive tumors [14]. Hanna et al. found that older patients were more likely to have positive margins and less likely to undergo re-excision due to comorbidities, leading to persistent positive margins [15]. Alves-Ribeiro et al. and Murphy et al. found no significant association between age, tumor markers, and margin status, reflecting the complexity of this issue [16,17].

This study has several limitations. First, its retrospective design introduces potential for selection bias, since the choice between Localizer and WL was not randomized but likely influenced by surgeon preference, lesion characteristics, and hospital logistics. Second, only univariate analyses were performed, meaning confounding factors, such as age, tumor type, or breast density, could not be adequately adjusted for. As a result, associations should be interpreted with caution. Third, the modest sample size and relatively low number of outcome events (positive margins, device migration) limit statistical power, raising the possibility of type II error and the probability that small but clinically relevant differences may not have been detected. Finally, reliance on a retrospective medical record review may have led to an underreporting of minor complications or margin details. Together, these biases restrict the strength of the conclusions, although they do not invalidate the findings.

## Conclusions

The method of localization, whether wire-guided or wireless, did not show statistically significant differences in terms of margin status or postoperative complications in this retrospective cohort. However, the Localizer offered greater flexibility in preoperative planning, as it can be inserted well in advance of surgery and is associated with shorter operative time. In addition, we identified two clinically relevant predictors of positive margins: older patient age and device migration. These findings, although secondary to the primary objective, provide actionable guidance for clinical practice by underscoring the importance of precise device placement and heightened surgical vigilance in older patients. Future research should first confirm, through larger prospective cohorts or randomized controlled trials, whether meaningful differences in surgical or oncologic outcomes exist between localization methods. Once clarified, subsequent studies should explore patient-centered outcomes, such as comfort, acceptability, and quality of life, to further inform best practice.

## References

[1] Heindl F, Schulz-Wendtland R, Jud S (2022). Evaluation of a wireless localization system for nonpalpable breast lesions - feasibility and cost-effectiveness in everyday clinical routine. In Vivo.

[2] Krebs in Deutschland für 2017/2018. 13 (2024). Cancer in Germany for 2017/2018 [In German]. Berlin.

[3] Institut National Du Cancer. (n.d (2024). Epidemiology and data in cancer [In French]. https://www.e-cancer.fr/Patients-et-proches/Les-cancers/Qu-est-ce-qu-un-cancer/Chiffres-cles.

[4] DiNome ML, Kusske AM, Attai DJ, Fischer CP, Hoyt AC (2019). Microchipping the breast: an effective new technology for localizing non-palpable breast lesions for surgery. Breast Cancer Res Treat.

[5] Dauphine C, Reicher JJ, Reicher MA, Gondusky C, Khalkhali I, Kim M (2015). A prospective clinical study to evaluate the safety and performance of wireless localization of nonpalpable breast lesions using radiofrequency identification technology. AJR Am J Roentgenol.

[6] Cullinane CM, Byrne J, Akmenkalne L (2021). The LOCalizer radiofrequency identification system: an effective new technology for localizing non-palpable breast lesions for surgery. Surg Innov.

[7] Malter W, Holtschmidt J, Thangarajah F, Mallmann P, Krug B, Warm M, Eichler C (2019). First reported use of the Faxitron LOCalizer™ radiofrequency identification (RFID) system in Europe - a feasibility trial, surgical guide and review for non-palpable breast lesions. In Vivo.

[8] Parisi S, Ruggiero R, Gualtieri G (2021). Combined LOCalizer™ and intraoperative ultrasound localization: first experience in localization of non-palpable breast cancer. In Vivo.

[9] Chan BK, Wiseberg-Firtell JA, Jois RH, Jensen K, Audisio RA (2015). Localization techniques for guided surgical excision of non-palpable breast lesions. Cochrane Database Syst Rev.

[10] Wang GL, Tsikouras P, Zuo HQ (2019). Radioactive seed localization and wire guided localization in breast cancer: a systematic review and meta-analysis. JBUON.

[11] McGugin C, Spivey T, Coopey S (2019). Radiofrequency identification tag localization is comparable to wire localization for non-palpable breast lesions. Breast Cancer Res Treat.

[12] The American Society of Breast Surgeons (2024). The American Society of Breast Surgeons. Breast cancer breast conservation surgery margins. Breast Cancer Breast Conservation Surgery Margins.

[13] Gooch JC, Yoon E, Chun J (2019). The relationship of breast density and positive lumpectomy margins. Ann Surg Oncol.

[14] Voguet L, Hébert T, Levêque J (2009). Patient age and positive margins are predictive factors of residual tumor on mastectomy specimen after conservative treatment for breast cancer. Breast.

[15] Hanna J, Lannin D, Killelea B, Horowitz N, Chagpar AB (2016). Factors associated with persistently positive margin status after breast-conserving surgery in women with breast cancer: an analysis of the National Cancer Database. Am Surg.

[16] Alves-Ribeiro L, Osório F, Amendoeira I, Fougo JL (2016). Positive margins prediction in breast cancer conservative surgery: assessment of a preoperative web-based nomogram. Breast.

[17] Murphy BL, Boughey JC, Keeney MG, Glasgow AE, Racz JM, Keeney GL, Habermann EB (2018). Factors associated with positive margins in women undergoing breast conservation surgery. Mayo Clin Proc.

